# A Tale of Two Lobsters—Transcriptomic Analysis Reveals a Potential Gap in the RNA Interference Pathway in the Tropical Rock Lobster *Panulirus ornatus*

**DOI:** 10.3390/ijms231911752

**Published:** 2022-10-04

**Authors:** Thomas M. Banks, Tianfang Wang, Quinn P. Fitzgibbon, Gregory G. Smith, Tomer Ventura

**Affiliations:** 1 Centre for Bioinnovation, University of the Sunshine Coast, Maroochydore, QLD 4556, Australia; 2 School of Science, Technology and Engineering, University of the Sunshine Coast, Maroochydore, QLD 4556, Australia; 3 Institute for Marine and Antarctic Studies (IMAS), University of Tasmania, Private Bag 49, Hobart, TAS 7001, Australia

**Keywords:** decapod crustaceans, achelata, RNA interference, gene silencing mechanism, transcriptomic analysis

## Abstract

RNA interference (RNAi) has been widely utilised in many invertebrate models since its discovery, and in a majority of instances presents as a highly efficient and potent gene silencing mechanism. This is emphasized in crustaceans with almost all taxa having the capacity to trigger effective silencing, with a notable exception in the spiny lobsters where repeated attempts at dsRNA induced RNAi have demonstrated extremely ineffective gene knockdown. A comparison of the core RNAi machinery in transcriptomic data from spiny lobsters (*Panulirus ornatus*) and the closely related slipper lobsters (*Thenus australiensis,* where silencing is highly effective) revealed that both lobsters possess all proteins involved in the small interfering and microRNA pathways, and that there was little difference at both the sequence and domain architecture level. Comparing the expression of these genes however demonstrated that *T. australiensis* had significantly higher expression in the transcripts encoding proteins which directly interact with dsRNA when compared to *P. ornatus*, validated via qPCR. These results suggest that low expression of the core RNAi genes may be hindering the silencing response in *P. ornatus*, and suggest that it may be critical to enhance the expression of these genes to induce efficient silencing in spiny lobsters.

## 1. Introduction

The discovery of RNA interference (RNAi) in the 1990s has greatly benefitted a wide variety of industries ranging from agriculture to therapeutics, and most pertinent here, aquaculture [1,2,3,4,5]. The ability to knock down genes discriminately and effectively granted by RNAi, can be used to generate desirable phenotypes, ranging from disease control, increased growth, timed reproduction and molting, and generation of monosex populations, while also functionally characterising genes of interest for particular aquaculture species [4].

### 1.1. Core RNAi Mechanism

The silencing effect of double-stranded (ds)RNA-induced RNAi was first discovered accidentally in petunias, and then specifically induced in *C. elegans*, leading to a significant revolution in molecular biology [1,6]. RNAi is now a popular choice in the toolkit of many molecular biologists, and works off the principle of specific gene silencing to modify gene expression at the mRNA level [7]. The term RNAi refers to a number of cellular processes, including the endogenous micro-RNA (miRNA) pathway, the antiviral/exogenous small interfering RNA pathway (siRNA), and the germline associated piwi interacting RNA pathway (piRNA), with the first two being the most relevant for this body of work [8,9]. The miRNA pathway begins with a pri-miRNA transcribed from the genome, which is processed into a pre-miRNA via the microprocessor complex consisting of the RNAse III Drosha and Pasha which binds dsRNA [10]. Pre-miRNAs are then exported through the nuclear pores into the cytoplasm, where Dicer 1 RNAse III enzymes cleave the pre-miRNA into mature miRNAs [11]. The mature miRNA then complexes with Argonaute 1 proteins and at least one dsRNA binding protein (such as TRBP, PACT, Nibbler, R2D2, etc.) to form the RNA induced silencing complex (RISC) [10,12,13,14,15,16,17]. The RISC then unwinds the RNA duplex and surveys the cell for a complementary mRNA sequence. Once a complementary mRNA sequence is detected, the RISC either degrades it or complexes with it and thereby blocks the mRNA translation [10].

The siRNA pathway has very similar core machinery but differs in the origin of a dsRNA substrate, which can be derived exogenously from viruses or the environment, or endogenously from transposons, and hybridisation of mRNA in cis and in trans (overlapping of the 3′ end in the sense and antisense transcript in the same gene, and complementary binding of sense and antisense of two separate genes, respectively) [18,19,20]. Dicer 2 then cleaves the dsRNA into 21–23 nt duplexes termed siRNAs, which are loaded into a RISC with Argonaute 2 and one or more dsRNA binding proteins (such as TRBP, C3PO, R2D2, PACT, etc.) and unwound before binding a complimentary mRNA and degrading it [15,17,21,22,23]. In vertebrates, a single dicer protein performs both the miRNA and siRNA cleavage, and one of four Argonaute proteins primarily cleaves mRNA [8,24]. In arthropods however, duplication events have led to distinct pathways with paralogous Dicers and Argonautes acting separately in the miRNA and siRNA pathways [25]. This mechanism is present in a vast majority of eukaryotes and possesses a dual role in endogenous gene regulation and antiviral defence [8,19,26,27]. The wide presence of RNAi in eukaryotes has enabled not only elucidation of new genes with reverse genetic techniques, but a suite of applications including, but not limited to, therapeutics and biomedicine, agriculture, aquaculture, and pest and biocontrol [2,3,28,29].

### 1.2. Dicer and Argonaute Domain Architecture

Despite gene duplications, deletions, and evolutionary pressure from viruses and viral silencing suppression in the siRNA pathway particularly, the core domains, and functions of Dicer and Argonaute proteins remain the most highly conserved RNAi components across metazoan evolution and are key to understanding the RNAi pathway [25,30]. At the N terminus, Dicer proteins possess highly conserved helicase domains which in arthropods, assist with substrate recognition [31]. The helicase of Dicer 1 proteins is unable to interact with ATP, allowing it to bind to pre-miRNAs without enacting unwinding activity, which enables the protein to ensure it is bound to the correct substrate prior to pre-miRNA cleavage [32,33]. Dicer 2 helicase inversely uses ATP to cleave long dsRNA into siRNAs [34]. The dsRNA binding protein R2D2 also assists in generating long dsRNA substrate specificity by inhibiting the ability of Dicer 2 to process and interact with pre-miRNA [34]. The domain of unknown function 283 (DUF283)/Dicer dimerization domain is positioned next to the helicase domains, and shows strong similarity to known dsRNA binding domains [35]. It is believed to facilitate binding with Dicer partners, and potentially assist in RNA remodeling and base pairing, though its function is largely uncharacterised [33,36]. The most important dicer domains are the PAZ and RNAse III domains, which act as the core of the protein and are largely responsible for the primary dicer functions of dsRNA binding and cleavage [33,37]. The PAZ domain contains 3′ and 5′ binding pockets which anchor in the dsRNA substrate and separate it from the RNAse III domains forming a ‘platform domain’ which structurally, leads to the generation of small ~22 nt siRNAs [38,39,40]. The RNAse III domains cleave each RNA strand leading to hydrolysis of the phosphodiester bond and generating 2–3nt overhangs with a 5′ phosphate and 3′ hydroxyl group at the cleavage site which is necessary for efficient silencing downstream in the RNAi pathway [33,38,41]. The dsRNA binding domain (dsRBD) at the C terminus was initially believed to have an auxiliary role in Dicer function but has since been shown to be important in broad recognition of dsRNA and for localisation of Dicer to the nucleus [42,43].

Argonaute proteins, like Dicers, consist of multiple functional domains which have various roles in RNA binding and processing, with an N, L1/DUF1785, PAZ, L2, MID, and PIWI domain from N terminus to C terminus [44]. The N domain is involved in passenger strand cleavage and unwinding for both miRNA and siRNA substrates which is critical for the formation of the RISC [45]. Interestingly, although it displays cleavage activity on miRNA/siRNA duplexes, N domain mutants showed no difference in degradation and catalysis of target mRNA suggesting this domain functions solely in the initiator steps of RISC directed gene silencing rather than the effector steps [45]. The Argonaute linker domains function to separate the other domains and enable the structural mechanics of the protein, but do not directly interact with RNA substrates [46,47]. Like Dicer, the Argonaute PAZ domain functions to anchor the 3′ end of the siRNA, while the MID domain anchors to lock the 5′ end in place [48,49,50]. This positions the siRNA to be bound to a complementary mRNA following unwinding, where the PIWI domain induces cleavage [51].

### 1.3. SID-1 dsRNA Channels and dsRNA Uptake

Of the two mechanisms of systemic RNAi thus far described, the dsRNA uptake mechanisms have been identified across more species than dsRNA transport mechanisms. The uptake of dsRNA occurs via dsRNA gated channels such as the systemic RNA interference defective protein 1 (SID-1) or through receptor-mediated endocytosis, with both mechanisms varying greatly across metazoan lineages [52,53,54,55,56,57,58]. SID-1 proteins are membrane-bound channels which passively transport RNA, independent of length, into the cell to be utilised via the core RNAi machinery, and are found across arthropod, nematode, and vertebrate lineages [54,59,60,61,62]. SID-1 proteins are characterized by multiple transmembrane domains and an extracellular domain involved in dsRNA recognition and binding, which preferentially binds longer dsRNA molecules [53,62]. These proteins also recognize and uptake siRNAs, however, the retention of siRNA is significantly less than that of long dsRNAs, which can be captured by RISC components such as Dicer and Argonaute, and thus do not escape the cell [59,63]. Uptake of dsRNA can also be facilitated through receptor mediated endocytosis, which has been displayed in both nematode and arthropod species [55,58,64,65]. In crustaceans, it was observed through pharmacological inhibition that receptor-mediated endocytosis is required for RNAi in some tissues, though no specific receptor has been described thus far, and further investigation is needed [65,66].

Among crustacean species used in aquaculture, there is little to no research surrounding the RNAi mechanism in the Achelata infraorder consisting of the economically important slipper lobsters and spiny lobsters, limiting the use of the technology in this area. These species show very different capacities for dsRNA induced silencing, with RNAi being accessible in the slipper lobster *Thenus australiensis* while inaccessible in the tropical rock lobster *Panulirus ornatus* [67]. In this study, we demonstrate that all the critical RNAi components are present in both lobster species but are significantly differentially expressed in the siRNA pathway between *T. australiensis* and *P. ornatus*. This disparity in availability of core RNAi machinery provides a mechanism to explain the limits for dsRNA induced silencing to occur in *P. ornatus* and provides a clear direction to induce silencing in this species.

## 2. Results

### 2.1. All Core RNAi Genes Are Present in Both P. ornatus and T. australiensis

**Figure 1:**
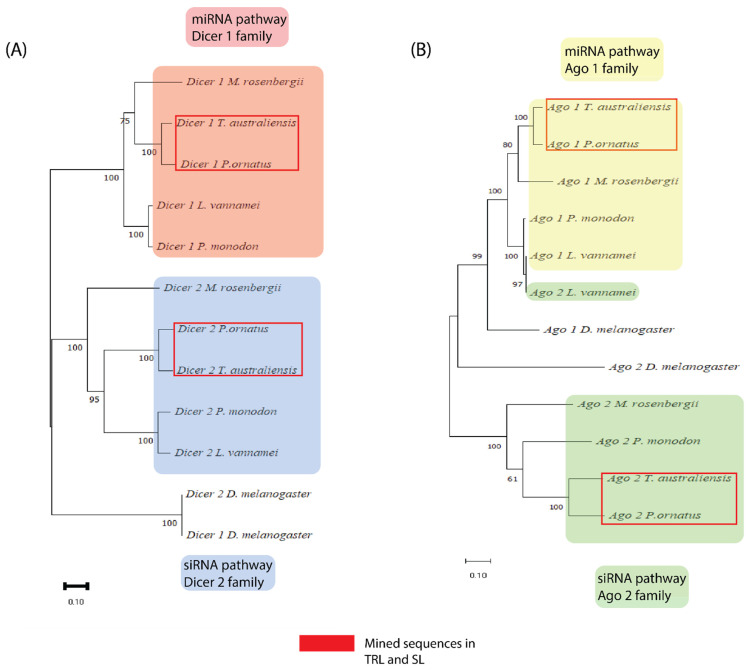
Maximum likelihood tree of putative amino acid sequences of Dicer proteins (**A**) and Argonaute proteins (**B**) from Panulirus ornatus and Thenus australiensis. Sequences for these genes were taken from crustaceans where the RNAi response is better studied (Macrobrachium rosenbergii, Penaeus monodon, and Litopenaeus vannamei) and used to predict the identity of putative transcripts taken from local *P. ornatus* and *T. australiensis* databases. The different coloured transparent boxes represent a different group of RNAi genes, and the red boxes represent the putative sequences mined from the local *P. ornatus* and *T. australiensis* databases.

Transcriptome mining with characterised RNAi genes from other arthropods (*L. vannamei*, *P. monodon*, *M. rosenbergii*, and *D. melanogaster*) revealed several putative RNAi gene transcripts. Two Dicer-like transcripts and two Argonaute-like transcripts were identified in both *P. ornatus* and *T. australiensis* with high inter and intraspecies sequence similarity. These sequences clustered in discrete groups with other crustacean orthologues (bootstrap > 95%) in a separate “Achelata” clade (Figure 1), with the exception of the Ago 1 transcripts which all clustered in one clade separate from *D. melanogaster* Ago 1 (Figure 1B). Apart from *L. vannamei* Ago 2 which clustered in the Ago 1 “super clade”, all other sequences ordered neatly into 4 discrete groups of crustacean genes with *D. melanogaster* as an outgroup in every case (Figure 1). This suggests that the sequences mined were firstly, distinct enough to be sorted into discrete groups which can indicate distinct function, and secondly, are homologs of RNAi genes and may therefore serve the same function as in other species. These Dicer and Argonaute transcripts alongside other RNAi genes were validated with a blastP search and domain architecture via SMART (Table 1). Every gene commonly associated with the miRNA and siRNA pathways were identified in both crustacean species (Table 1).

**Figure 2:**
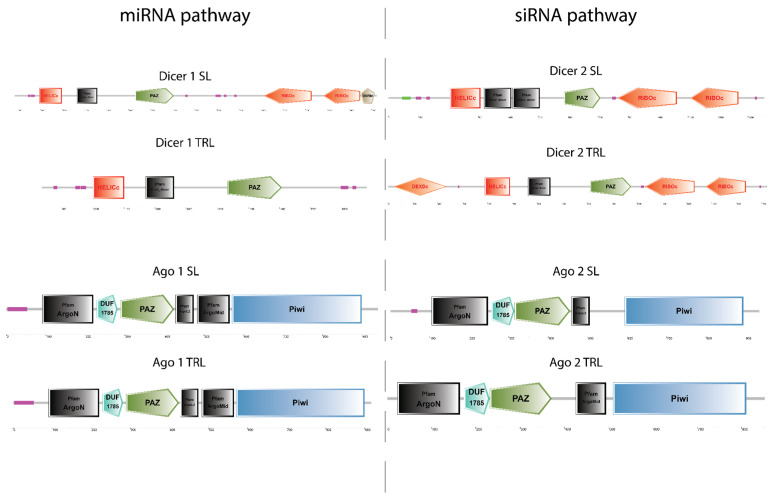
Domain architecture across core small interfering RNA (siRNA) and microRNA (miRNA) pathways in the slipper lobster *Thenus australiensis* (SL) and the ornate tropical rock lobster *Panulirus ornatus* (TRL). Architecture was resolved via SMART in NORMAL mode using miRNA gene sequences (Dicer 1 and Ago 1) and siRNA gene sequences (Dicer 2 and Ago 2) from both species. Purple/pink boxes represent regions of low complexity, black domains represent domains from the PFAM database, and all others are from the SMART database.

Following phylogenetic analysis to validate the identity of the putative RNAi genes, sequences were subjected to a domain architecture search via SMART. For the Dicer 1 sequences, the only difference in domain architecture is that the *T. australiensis* Dicer 1 has two RIBOc domains and one DSRM which are missing from the *P. ornatus* Dicer 1 (Figure 2). This may be simply a result of the RNA-seq assembly truncating the gene, rather than an actual biological and functional difference. For Dicer 2, all domains were found in both species, with the exception of one helicase domain in *T. australiensis*, however when performing a domain architecture search of the N terminal region, the DEXDc domain appears. This variation may be a result of the domains partially overlapping which prevents the SMART algorithm from accurately discerning two distinct domains. For Argonaute 1, there was no difference in domain architecture between *T. australiensis* and *P. ornatus* suggesting high conservation (Figure 2). In Argonaute 2, the only difference was *T. australiensis* had an ArgoL2 domain while *P. ornatus* had an ArgoMid domain (Figure 2). This difference could be attributed to the transcriptome assembly generating slightly overlapping domains which leads to the SMART output showing a single domain instead of both the ArgoMid and ArgoL2 domains.

### 2.2. Variable Expression of siRNA and miRNA Genes in P. ornatus across Metamorphic Stages

As both *P. ornatus* and *T. australiensis* possess the core RNAi machinery, and silencing has been induced in slipper lobsters but not spiny lobsters, the expression of the genes involved in the siRNA and miRNA pathways was analysed in both species [67]. For *P. ornatus*, we had access to a comprehensive transcriptome library including multiple life stages, and multiple adult tissues [68,69]. In *T. australiensis* however, there is only one dataset produced recently (unpublished data) which contains the sub adult tissues, making direct comparison difficult between both species.

**Figure 3:**
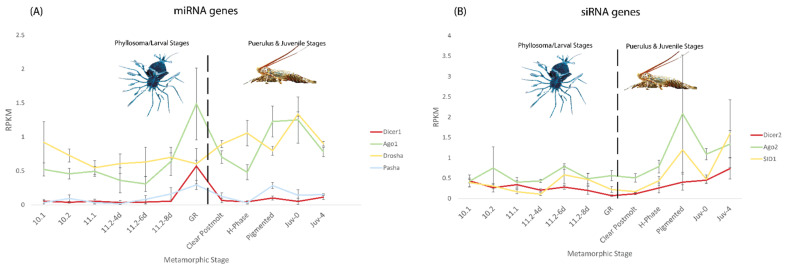
Micro RNA (miRNA) pathway (**A**) and small interfering RNA (siRNA) pathway (**B**) gene expression across metamorphic stages of the ornate lobster Panulirus ornatus (n = 3 for each stage). Gene expression is measured in reads per kilobase per million reads (RPKM), and stages left of the broken line (10.1, 10.2, 11.1, 11.2-4D, 11.2-6D, 11.2-8D, and gut retraction) represent the larval/phyllosoma stages, while those right of the broken line (clear postmolt, H-phase, pigmented, Juv-0, and Juv-4) represent the puerulus and juvenile stages. In (**A**) Dicer 1 is displayed in red, Argonaute 1 in green, Drosha in yellow, and Pasha in blue. In (**B**) Dicer 2 is displayed in red, Argonaute 2 in green, and SID1 in yellow.

Across the metamorphic stages of *P. ornatus* (described in Hyde et al. (2019)), expression of genes in both the siRNA and miRNA pathways were variable and appear to change significantly based on life stage, however overall, expression was very low (≤2.0 RPKM; Figure 3) [68]. In the miRNA related genes, Dicer 1 and Ago 1 both peaked in expression at the gut retraction stage with relatively stable levels of expression preceding this spike (Figure 3B). After the peak, Dicer 1 expression dropped back to very low levels (RPKM < 0.2), while Argonaute 1 dropped in expression, then rose again in the pigmented and Juv-0 stage before dropping again in the Juv-4 stage (Figure 3A). Drosha had relatively consistent expression until after the gut retraction stage where it began to rise slowly before decreasing towards the Juv-4 stage (Figure 3A). Pasha like Dicer 1 had very poor expression overall with a slight peak at gut retraction and an additional smaller peak at the pigmented stage (Figure 3A). 

In the siRNA pathway, all three genes trended upward in expression towards the juvenile stages rather than the larval stages. Ago2 and SID1 both had a sharp peak at the pigmented stage and expressed higher in the Juv-0 and Juv-4 stages than in the earlier stages (Figure 3B). A similar pattern existed for Dicer 2, however without the peak at the pigmented stage (Figure 3B). Despite the relatively high levels of expression towards the juvenile stages of these genes, the RPKM values were low and thus may not translate to highly expressed and active genes in situ.

### 2.3. Core RNAi Machinery Is Expressed Significantly Higher in T. australiensis Than P. ornatus across Adult and Sub-Adult Tissues

**Figure 4:**
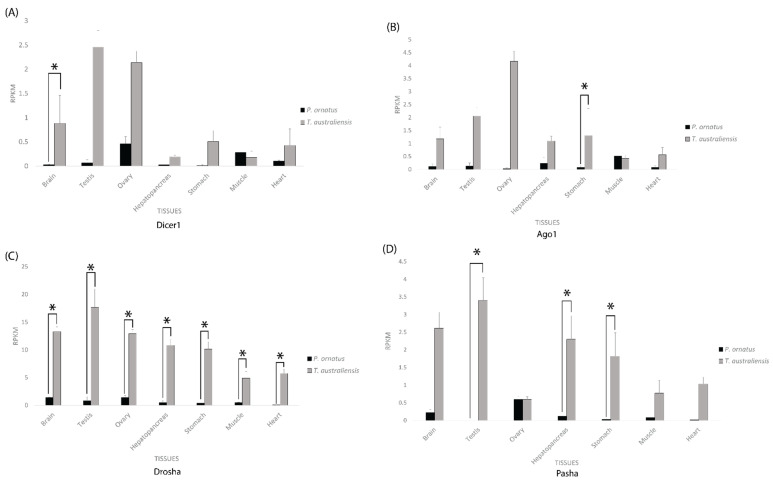
Expression of micro RNA (miRNA) pathway genes across adult tissues in *Panulirus ornatus* (n = 3) and *Thenus australiensis* (n = 3). Genes include Dicer 1 (**A**), Argonaute 1 (**B**), Drosha (**C**), and Pasha (**D**). Expression of genes is measured in reads per kilobase per million reads (RPKM). A black bar represents tissues from *P. ornatus*, and a grey bar represents tissues from *T. australiensis*. Tissues from left to right include brain, testis, ovary, hepatopancreas, stomach, muscle, and heart. An asterisk represents a statistically significant (*p* < 0.05) difference in expression.

**Figure 5:**
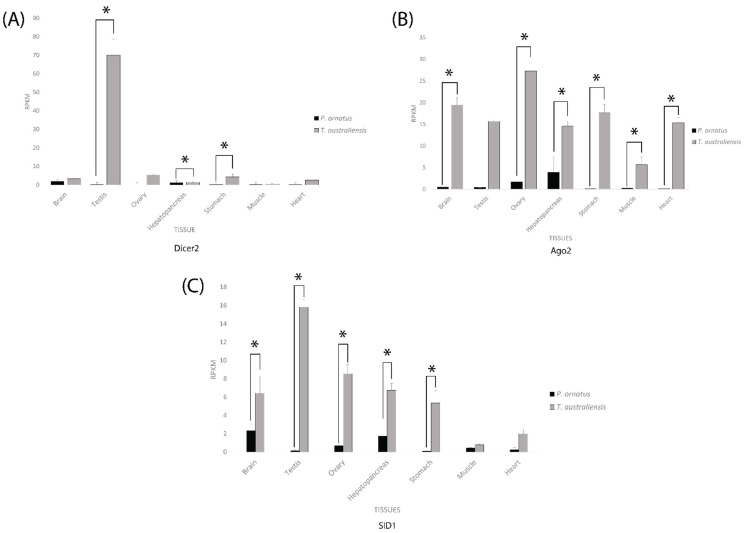
Expression of small interfering RNA (siRNA) pathway genes (**A**-Dicer 2, **B**-Argonaute 2, **C**-SID1) across adult tissues in *Panulirus ornatus* (n = 3) and *Thenus australiensis* (n = 3). Expression is measured in reads per kilobase per million reads (RPKM). Black bar represents tissues from *P. ornatus*, grey bar represents tissues from *T. australiensis*. Tissues from left to right are brain, testis, ovary, hepatopancreas, stomach, muscle, and heart. An asterisk represents a statistically significant (*p* < 0.05) difference in expression.

In the tissues of both adult lobster species, across both the miRNA and siRNA pathways, gene expression was generally higher in *T. australiensis* than *P. ornatus*, with a few minor exceptions of approximately equal expression in the brain (Dicer 2), ovary (Pasha), hepatopancreas (Dicer 1 and 2), and muscle (Dicer 1 and 2, Ago 1, and SID-1) (Figure 4 and Figure 5). 

Across the miRNA pathway, *T. australiensis* Dicer 1 showed higher expression than *P. ornatus* Dicer 1 in all tissues except for the muscle where the latter showed slightly greater levels of expression, yet only the brain showed a significant difference (Figure 4). The same trend was observed for Ago 1, except that only the stomach showed statistically significant difference (Figure 4). *T. australiensis* Drosha showed consistently higher expression than *P. ornatus* Drosha and was statistically significant across all tissues. A similar trend was observed for Pasha in both species, except only the testis, hepatopancreas, and stomach had significant differences in expression, and the ovary had approximately equal expression (Figure 4). Overall *T. australiensis* possessed the greatest levels of expression compared to *P. ornatus* across most tissues and genes. Across the siRNA pathway, *T. australiensis* consistently showed significantly higher expression in the hepatopancreas and stomach than *P. ornatus* while the other tissues were usually significantly higher as well, but not consistently across all samples (Figure 4). This difference was not as pronounced in Dicer 1 and Ago 1 in the miRNA pathway but was evident for Drosha and Pasha (Figure 4). 

For the siRNA related genes, *T. australiensis* Dicer 2 had extremely high expression in the testis compared to the rest of the tissues and was significantly higher than *P. ornatus* testis (Figure 5). In the brain, muscle, and hepatopancreas there was relatively similar RPKM for both species, with a statistically significant difference in the hepatopancreas. The ovary, stomach, and heart, all showed higher expression for *T. australiensis* Dicer 2 than *P. ornatus* Dicer 2, however only the stomach was statistically significant (Figure 5). For Ago 2, *T. australiensis* had higher expression than *P. ornatus* in every tissue with all tissues except the testis displaying a significant (I < 0.05) difference (Figure 5). A similar trend was seen in SID1, except for the muscle tissue samples which were not considerably different between *P. ornatus* and *T. australiensis*. Despite this, only the brain, ovary, hepatopancreas, and stomach showed a statistically significant difference.

### 2.4. dsRNA Exposure Does Not Modulate siRNA Pathway Expression in P. ornatus and T. australiensis

**Figure 6:**
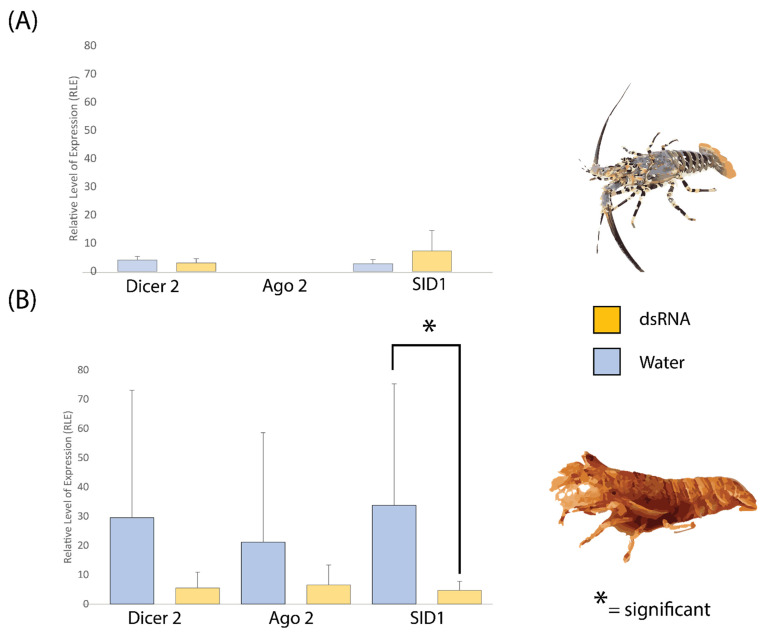
Expression relative to 18S of Dicer 2, Argonaute 2, and SID1 in Panulirus ornatus (TRL; **A**) and Thenus australiensis (**B**; SL) in the hepatopancreas 24 h following injection with either filtered seawater or non-specific double stranded RNA (dsRNA; dsGFP sequence homologous to green fluorescent protein). An asterisk denotes a significant difference in expression.

The siRNA pathway is directly responsible for interacting with dsRNA to cause silencing phenotypes, and we observed that *P. ornatus* displayed consistently poor expression of the major pathway components (Figure 5). Previous literature indicates that this pathway can be induced via dsRNA exposure, which was investigated here with qPCR [70]. In both *P. ornatus* and *T. australiensis,* dsRNA injection had no significant effect on Dicer 2, Argonaute 2, or SID1 expression in the hepatopancreas, except for *T. australiensis SID1* which was significantly lower in the animals injected with dsRNA (Figure 6). In *P. ornatus* however, expression of these genes relative to 18S was low compared to *T. australiensis*, with Argonaute 2 being completely undetectable (Figure 6). This is consistent with transcriptomic data and further supports the poor siRNA gene expression in *P. ornatus* (Figure 5 and Figure 6). 

## 3. Discussion

In a vast majority of crustacean models, dsRNA induced gene silencing is highly potent and effective [17,28,70,71,72,73]. Notably absent from this group are the spiny lobsters, where repeated attempts at silencing in multiple species have clearly demonstrated the inability of this taxa to generate RNAi phenotypes (personal communication). The closest relative of the spiny lobsters (family Palinuridae) are the slipper lobsters (family Scyllaridae, from the same infraorder, achelata), where silencing has been shown to be highly effective, prompting a comparison of the silencing mechanisms between these species [67]. Across the miRNA and siRNA pathways, both *P. ornatus* and *T. australiensis* possessed all the core machinery necessary to elicit silencing (Figure 1, Table 2). Further supporting this is the fact that Dicer 2 and Argonaute 2 in both species had correct domain architecture, which indicates that the RNAi machinery is functioning correctly, but either is expressed poorly or unable to interact with dsRNA due to another mechanism. Across the life stages of *P. ornatus*, expression of the siRNA pathway was consistently low, which indicates there is likely no ‘best time’ to silence. This is contrary to several insect groups, where, for example in lepidopterans, the larval stages are notoriously recalcitrant to RNAi while the pupal and adult stages are more receptive to silencing [74,75]. This may indicate that larvae of lepidopterans may also have poor expression of the siRNA pathway similar to *P. ornatus* in addition to the other described mechanisms preventing RNAi in these species [76,77]. In the case of the miRNA pathway which plays a critical role in various biological functions, Dicer 1 and Pasha were seen to have extremely low expression (Figure 3) [78]. This indicates that *P. ornatus* may primarily use a Dicer 1 independent miRNA biogenesis pathways, which, coupled with a low turnover of Drosha and Argonaute 1, could enable a functional miRNA pathway despite the low transcriptomic expression (Figure 3) [79]. In mammalians, Drosha and Argonaute can generate mature miRNAs independent of Dicer, which could be occurring more frequently in *P. ornatus* to combat low Dicer 1 expression [78,80]. Dicer 1 also has a small peak at the gut retraction life stage where it could be recruited right before the animal metamorphoses, which represents a large developmental shift, to generate more miRNAs which might be required to enable the anatomical and physiological changes (Figure 3).

When comparing the tissues of *P. ornatus* and *T. australiensis*, significant differences in the expression of the siRNA and miRNA pathway were observed (Figure 4 and Figure 5). Across the miRNA pathway, only Drosha displayed significantly higher expression in *T. australiensis* compared to *P. ornatus* consistently across all tissues (Figure 4). Drosha has been implicated in other pathways outside RNAi including but not limited to transcriptional activation, alternative splicing regulation, mRNA degradation, and antiviral defence, so its higher expression in *T. australiensis* may be related to these processes rather than the miRNA pathway [81]. For Dicer 1, Argonaute 1, and Pasha, *T. australiensis* in general had higher expression than *P. ornatus* across tissues, but this was rarely significant and occurred across a small RPKM range (Figure 4). This is consistent with the miRNA pathway expression across life stages in *P. ornatus*, and may imply similarly that a consistent expression and low turnover rate of these proteins may be enough to keep the pathway functioning in *T. australeinsis* as well (Figure 3). 

With a few exceptions, every siRNA related gene in *T. australiensis* had significantly higher expression across multiple tissues when compared to *P. ornatus* (Figure 5). This is consistent with the silencing capacity of these species, and offers a mechanism to explain the disparity between spiny lobster and other crustacean RNAi [67]. A low basal siRNA pathway expression in *P. ornatus* would limit the potential for dsRNA to be uptaken into the cell and processed into siRNAs for RISC assembly and inhibit the subsequent silencing. Similarly, the greater expression levels of the siRNA pathway components in *T. australiensis* would likely lead to a greater silencing response. Previous work identified a lack of dsRNA transport proteins in spiny lobster hemolymph, and this coupled with very poor expression of the core RNAi machinery could hinder silencing in the systemic and intracellular sense [67]. As the siRNA pathway is one of the primary antiviral mechanisms in arthropods, there is only one known spiny lobster virus, and *P. ornatus* displays consistently poor expression of this pathway, there is scope to speculate these lobsters may have evolved different means to combat viral infection outside of RNAi [18]. These alternative antiviral pathways may also be interfering with silencing induction, as dsRNA molecules act as a viral pathogen-associated molecular pattern (PAMP) which may be targeted by these immune mechanisms, thus potentially inhibiting RNAi.

Following dsRNA exposure, there were almost no significant differences in the siRNA pathway genes in the hepatopancreas for both *P. ornatus* and *T. australiensis* (Figure 6), unlike that in *M. rosenbergii*, where dsRNA pre-exposure greatly increased the expression of Dicer 2, Argonaute 2, and SID1, but not miRNA associated proteins [70]. This suggests a very specific mechanism to enhance the antiviral RNAi system in response to a dsRNA PAMP, which is seemingly absent in *T. australiensis* and *P. ornatus*. In the case of *P. ornatus*, this is unsurprising given that the species consistently shows a poor response to RNAi, and may represent the need to promote dsRNA entry into cells by the formation of dsRNA nanoparticles or riboprotein complexes (personal communication) [67]. For *T. australiensis*, where silencing was shown to be highly effective like that of *M. rosenbergii*, it was expected that the siRNA pathway would be greatly upregulated in a similar manner, however dsRNA injection did not modulate Dicer 2 or Argonaute 2, and actually inhibited SID1 expression [28,67]. This lack of upregulation may imply that the hepatoprancreas is unreceptive to dsRNA modulation in *T. australiensis* while other tissues may be, or that other confounding variables such as viral infection and life history may be hindering this response. In the case of SID1, *L. vannamei* SID1 is required for dsRNA uptake in the gills, but dsRNA uptake in the hepatopancreas is mediated by receptor endocytosis. A similar mechanism may occur in *T. australiensis* and could explain why dsRNA administration suppressed SID1 expression to reduce competition with a dsRNA specific receptor. dsRNA uptaken via receptors may be processed differently to enable systemic spread as the hepatopancreas is the interface between the animal and the environment, so uptake via SID1 may be detrimental to this process. Across the qPCR data there was great variability in expression of the siRNA pathway however, with individuals being consistent across all three genes, i.e., those with the highest expression for Dicer 2 also had the highest levels of expression for Argonaute 2 and SID1 (see Supplementary Materials, Table S1). This may be indicative of population specific factors or perhaps previous viral exposure which may either promote or impede RNAi by modulating siRNA pathway expression [82]. 

Overall, relative to 18S at least, *T. australiensis* displays far greater expression of the siRNA pathway than *P. ornatus* which is consistent with transcriptomic data. This is emphasized in particular by *P. ornatus* Argonaute 2, which across both treatments was undetectable, suggesting very poor expression. This lack of expression of the siRNA pathway components, like that in the transcriptome, would prevent efficient silencing following dsRNA injection, and likely must be enhanced to allow for RNAi to occur.

## 4. Materials and Methods

### 4.1. RNAi Gene orthologue Collection

Genes canonically involved in the miRNA and siRNA pathway were identified in the literature to be used for transcriptome mining in *P. ornatus* and *T. australiensis*. Sequences from decapod species were retrieved from the NCBI GenBank databases. These include Dicer 1 (KY369130.1), Dicer 2 (KY369131.1), Argonaute 1 (KY369127.1), Argonaute 2 (KY369128.1) and SID1 (ASU89918.1) from *Macrobrachium rosenbergii*, as well as Drosha (ROT62179.1), Pasha (HQ692889.1), and TRBP (XP_027225502.1) from *Litopenaeus vannamei*.

### 4.2. Transcriptome Mining in P. ornatus and T. australiensis and SMART Annotation of RNAi Genes

RNAi gene orthologues were submitted to a tBLASTN search on CrustyBase (https://crustybase.org/blast/, accessed on 4 July 2022) utilising both the metamorphic stages and adult tissues databases for *P. ornatus* and the multiple sub-adult tissues database for *T. australiensis* [68,69,83]. The most complete sequences with e-values of <1.0 × 10^−30^ and >50% query coverage, were selected for further analysis. Transcripts were translated with Expasy (https://web.expasy.org/translate/, accessed on 4 July 2022), and putative amino acid sequences were subjected to a BLASTP search using the NCBI non-redundant protein database and a domain architecture search via SMART (http://smart.embl-heidelberg.de/smart/set_mode.cgi?NORMAL=1, accessed on 4 July 2022) to predict identity [84,85]. 

### 4.3. Phylogenetic Analysis of Dicer and Argonaute Genes

Dicer 1 and 2 and Argonaute 1 and 2 transcripts possessed high sequence similarity to each other, respectively, and were thus resolved via phylogeny. Orthologues of these genes from other arthropods (which have been previously characterised, including *L. vannamei*, *Penaeus monodon*, *M. rosenbergii*, and *Drosophila melanogaster*) were used to resolve these ambiguous transcripts. The amino acid sequences were aligned with ClustalW on the MEGA11 platform with default settings, and a maximum likelihood tree (with 1000 bootstrap replicates) was constructed using default settings and the WAGS + F substitution model.

### 4.4. Transcriptome Quantification and Tissue Specific RNAi Expression

Quantification of the transcriptomes for *P. ornatus* and *T. australiensis* were performed previously as described in Ventura et al. (2020) and Hyde et al. (2019) [68,69]. These expression data from both *P. ornatus* and *T. australiensis* were used to graph reads per kilobase per million reads (RPKM) of miRNA and siRNA genes in a tissue specific manner. In the cases where tissues had multiple replicates for a certain stage or sex, the data were averaged for direct comparison between *P. ornatus* and *T. australiensis*. Statistical analysis was performed on these samples using student’s t test after FDR correction, with a *p*-value of <0.05 denoting statistical significance.

### 4.5. RNAi Modulation, RNA Extraction, and RT qPCR

To assess the capacity for RNAi modulation, *P. ornatus* and *T. australiensis* juvenile individuals (20–50 g animals) were injected with either 5 µg/g animal of ds*GFP* (dsRNA homologous to green fluorescent protein; n = 6) or equivalent volume of filtered seawater (n = 6) in the 5th walking leg sinus. The hepatopancreas was then dissected 24 h post injection, and RNA extraction was performed using RNAzol^®^ RT (Molecular Research Center; Cincinnati, OH, USA) supplemented with 1% ß-mercaptoethanol as described previously [68]. RNA was then dissolved in 50 µL DEPC treated water followed by assessment of quality and yield via nanodrop (yield: 160–1000 ng/µL, A260/230: 1.4–2.5, A260/A280: 1.9–2.1) prior to storage at stored at −80 °C. Following this, 1µg of RNA was reverse transcribed to cDNA using the Tetro cDNA synthesis kit (Meridian Bioscience; Cincinnati, OH, USA), and the expression of Dicer 2, Argonaute 2, and SID1 were quantified for both species via qPCR, with 18S being used to normalise expression (see S1 for primers). Primers were designed using an online tool to simulate the Assay Design Center from Roche (https://primers.neoformit.com/, accessed on 4 July 2022) and the relevant probe was used for each assay (Table 2) Reactions were performed in the Rotor Gene 6000 thermocycler, and relative gene expression was determined using the 2^−ΔΔCT^ method. To determine statistically significant differences between treatments, an ANOVA test was used followed by a Kruskal–Wallis non-parametric test.

## 5. Conclusions

In this study, the RNAi pathways of two closely related lobster species with significantly different capacities for RNAi induced gene silencing were investigated. It was shown that all RNAi components are present in both *P. ornatus* and *T. australiensis*, which should enable potent silencing in both lobster species, however transcriptomic analysis revealed significant differences in gene expression. Across the siRNA pathway which directly interacts with exogenous dsRNA for gene silencing, *T. australiensis* displayed consistently greater expression across the core machinery than *P. ornatus* across tissues. This suggests that, while the pathway is active in both lobster species, only *T. australiensis* expresses it highly enough to trigger detectable/significant silencing. Surprisingly, dsRNA exposure did not modulate the siRNA pathway in either lobster species as previously described in other arthropods, but qPCR analysis of these genes again revealed more robust expression in *T. australiensis* than *P. ornatus*, which is consistent with transcriptomic data. These results highlight the importance of RNAi pathway expression as a means of inducing potent silencing and suggest a clear direction forward in enabling silencing in *P. ornatus* by identifying methods to upregulate the siRNA pathway.

## Figures and Tables

**Table 1:** RNA interference genes identified in *T. australiensis* and *P. ornatus*, which pathway they belong to, the best blast hit from NCBI non-redundant protein database, and e-value for these blast results.

Gene Name	*Thenus australiensis*	*Panulirus ornatus*	RNAi Pathway	Best Blast Hit (NCBI)	E Value
Dicer1	✓	✓	miRNA	endoribonuclease Dcr-1-like [*Homarus americanus*]	0
Dicer2	✓	✓	siRNA	endoribonuclease Dicer-like [*Homarus americanus*]	0
Ago1	✓	✓	miRNA	argonaute 1 [*Panulirus interruptus*]	0
Ago2	✓	✓	siRNA	protein argonaute-2-like [*Homarus americanus*]	0
Drosha	✓	✓	miRNA	LOW QUALITY PROTEIN: ribonuclease 3-like [*Penaeus monodon*]	0
Pasha	✓	✓	miRNA	microprocessor complex subunit DGCR8-like [*Homarus americanus*]	0
TRBP	✓	✓	miRNA and siRNA	RISC-loading complex subunit tarbp2-like [*Homarus americanus*]	0
SID1	✓	✓	siRNA	uncharacterized protein LOC121864209 isoform X1 [*Homarus americanus*]	0

**Table 2:** List of primers used for qPCR and respective species.

Species	Gene Name	Forward Primer Sequence (5′–3′)	Reverse Primer Sequence (5′–3′)	Amplicon Size	Probe Used
*Thenus australiensis*	Dicer2	TCATAACCGTCAGCAACCCA	GGGCCCTCACATCCATAAGG	96	55
	Ago2	TTAACCATCCACCTGCAGGC	GCGTACCTGTCCATAGAGGC	73	58
	SID1	GGGGAAACGGAAGGAATGGA	GCATGTTGGGGTCATCCTCA	79	120
	18S	GGTGCATGGCCGTTCTTA	TGGAGATCCGTCGACTAGTTAAT	94	22
*Panulirus ornatus*	Dicer2	GGGCACATGAACCTGGTACA	GAAGCTCTTTGTTCGGTCGC	129	12
	Ago2	CAAGAACGGGGGATGACCAT	TCTGGCAAATCTCCCTCTGG	77	151
	SID1	TTTGCTGCCCTACCTACTGC	AAGCACCGATCCTCAACTCC	80	89
	18S	AACGGACTTGACGGTTGGTT	CTGTTCGGAGCCTGACAGAA	70	49

## Data Availability

Transcriptomic data from -*P. ornatus* and *T. australiensis* can be accessed from https://crustybase.org, accessed on 4 July 2022.

## Supplementary Materials

The following supporting information can be downloaded at: https://www.mdpi.com/article/10.3390/ijms231911752/s1.
